# A Brief Report on an Implantation of Small-Caliber Biodegradable Vascular Grafts in a Carotid Artery of the Sheep

**DOI:** 10.3390/ph13050101

**Published:** 2020-05-21

**Authors:** Larisa V. Antonova, Andrey V. Mironov, Arseniy E. Yuzhalin, Evgeniya O. Krivkina, Amin R. Shabaev, Maria A. Rezvova, Vadim O. Tkachenko, Mariam Yu. Khanova, Tatiana Yu. Sergeeva, Sergei S. Krutitskiy, Leonid S. Barbarash

**Affiliations:** 1 Research Institute for Complex Issues of Cardiovascular Diseases, 6 Sosnovy Blvd, Kemerovo 650002, Russia; antonova.la@mail.ru (L.V.A.); a.mir.80@mail.ru (A.V.M.); leonora92@mail.ru (E.O.K.); neirohirurgi@yandex.ru (A.R.S.); rezvovamaria@mail.ru (M.A.R.); Khanovam@gmail.com (M.Y.K.); sergtu@kemcardio.ru (T.Y.S.); kss911@mail.ru (S.S.K.); reception@kemcardio.ru (L.S.B.); 2 Budker Institute of Nuclear Physics SB RAS, 11 akademika Lavrentieva Ave, Novosibirsk 630090, Russia; vtkachen@mail.ru

**Keywords:** biodegradable vascular graft, polycaprolactone, carotid artery implantation, sheep, pre-clinical study, heparin, iloprost, VEGF, bFGF, SDF-1α

## Abstract

The development of novel biodegradable vascular grafts of a small diameter (<6 mm) is an unmet clinical need for patients requiring arterial replacement. Here we performed a pre-clinical study of new small-caliber biodegradable vascular grafts using a sheep model of carotid artery implantation. The 4 mm diameter vascular grafts were manufactured using a mix of polyhydroxybutyrate/valerate and polycaprolactone supplemented with growth factors VEGF, bFGF and SDF-1α (PHBV/PCL-GFmix) and additionally modified by a polymer hydrogel coating with incorporation of drugs heparin and iloprost (PHBV/PCL-GFmix^Hep/Ilo^). Animals with carotid artery autograft implantation and those implanted with clinically used GORE-TEX^®^ grafts were used as control groups. We observed that 24 h following surgery, animals with carotid artery autograft implantation showed 87.5% patency, while all the PHBV/PCL-GFmix and GORE-TEX^®^ grafts displayed thrombosis. PHBV/PCL-GFmix^Hep/Ilo^ grafts demonstrated 62.5% patency 24 h following surgery and it had remained at 50% 1 year post-operation. All the PHBV/PCL grafts completely degraded less than 1 year following surgery and were replaced by de novo vasculature without evidence of calcification. On the other hand, GORE-TEX^®^ grafts displayed substantial amounts of calcium deposits throughout graft tissues. Thus, here we report a potential clinical usefulness of PHBV/PCL grafts upon their additional modification by growth factors and drugs to promote endothelialization and reduce thrombogenicity.

## 1. Introduction

Cardiovascular disease resulting from atherosclerosis is a leading cause of death worldwide [1]. Treatment of established disease includes surgical resection of affected vessels following implantation of a vascular graft. Autologous blood vessels used as autografts are the best option for such surgery; however, their availability is frequently limited and harvesting such vessels is associated with risk of complications [2,3]. Currently available artificial prostheses with a diameter of <6 mm tend to develop thrombosis and neointimal hyperplasia in the long-term [4,5]. To improve vascular disease management, there is a need to develop novel tissue-engineered small-diameter vascular grafts of better functionality and reliability.

In recent years, a number of strategies have been proposed to enhance synthetic vascular grafts in order to promote their endothelialization by the host. Most of these approaches include immobilization of cell adhesion proteins and bioactive peptides on the luminal surface of the graft [6,7]. Such functionally active biodegradable vascular grafts potentiate the replacement of a polymer backbone with a new vessel [8,9,10]. Accumulating evidence from experimental studies shows that polymer grafts supplemented by bioactive molecules are beneficial over standard unmodified grafts [11,12].

Previously, our group developed a small caliber electrospun biodegradable graft based on a polyhydroxybutyrate/valerate (PHBV) and polycaprolactone (PCL) blend, additionally modified by various pro-angiogenic factors [13,14]. Using a rat model of abdominal aorta implantation, we showed that immobilization of angiogenic factors on graft luminal surface led to a rapid repopulation of PHBV/PCL grafts by endothelial progenitor cells [14,15]. Three months following implantation in rats, we observed a complete re-endothelialization along with formation of neointima and the presence of smooth muscle cells producing collagen types I and IV [14,15]. The primary patency of such grafts was 93.3% 12 months following implantation.

Based on these successful preliminary findings, we conducted a pre-clinical trial to examine the patency of PHBV/PCL grafts modified by growth factors VEGF, bFGF, stromal cell-derived factor 1 (SDF-1α), antiplatelet agents and anticoagulants, upon the implantation into the carotid artery of the sheep. 

## 2. Results 

**Figure 1:**
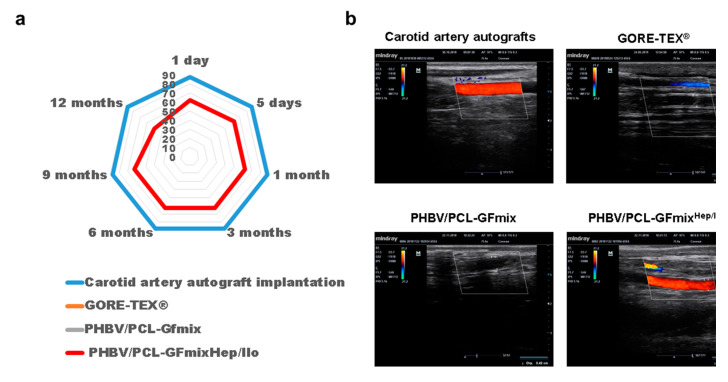
Ultrasonography studies of sheep implanted with experimental vascular grafts. (**a**) Patency of vascular grafts (%) at different timepoints following implantation into the carotid artery of sheep. Note that GORE-TEX^®^ and PHBV/PCL-GFmix are not shown because their patency was 0% during the entire study. (**b**) Representative ultrasonography images of sheep’ carotid arteries after graft implantation. Red areas indicate the blood flow.

**Figure 2:**
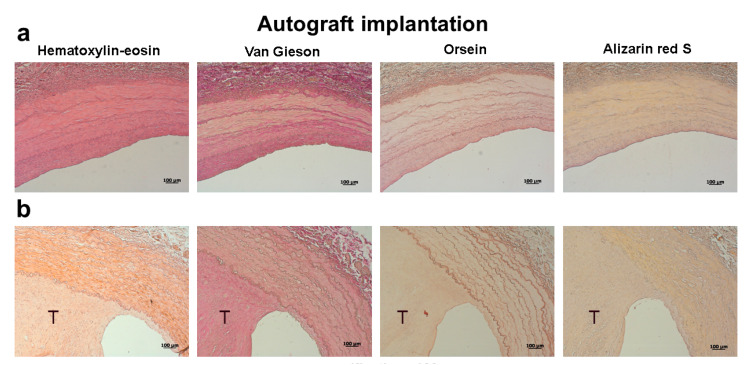
Histological examination of carotid artery autografts explanted 12 months following surgery. (**a**) Fully patent carotid artery. (**b**) Thrombotic carotid artery. The thrombus is indicated by the letter T. Scale bar = 100 μm.

Seven out of eight sheep (87.5%) implanted with carotid artery autografts demonstrated a complete patency the next day after surgery, and this number did not change during the 12 months of the experiment (Figure 1, Figure 2a). In only one animal, a parietal thrombus was detected the next day following surgery (Figure 2b).

**Figure 3:**
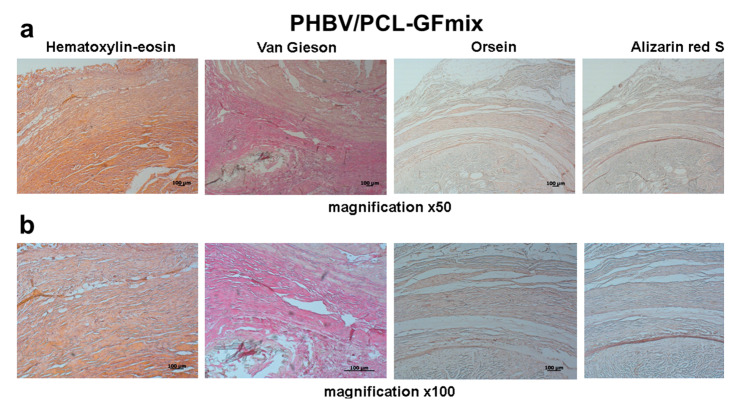
Histological examination of PHBV/PCL-GFmix grafts explanted 12 months following surgery. (**a**) Magnification 50×. (**b**) Magnification 100×. Scale bar = 100 μm.

**Figure 4:**
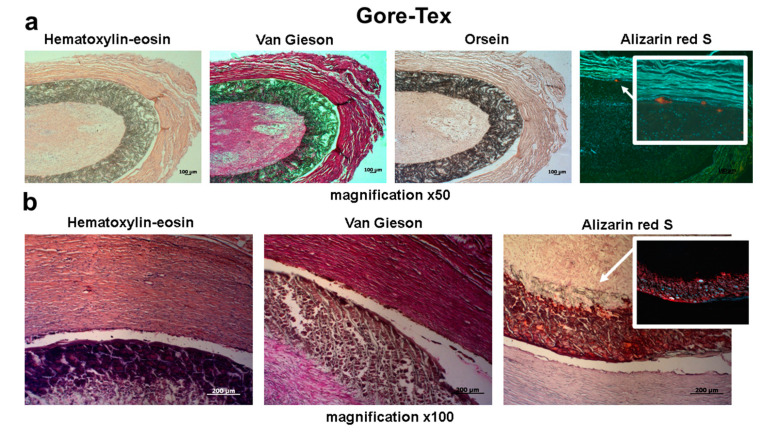
Representative images of GORE-TEX^®^ grafts explanted 6 months following surgery. Alizarin red S staining showed for both bright field and fluorescence microscopy. (**a**) Representative sample with a single calcium deposit. (**b**) Representative sample with massive deposition of calcium in the graft wall. Scale bar = 100 μm.

Both PHBV/PCL-GFmix and GORE-TEX^®^ grafts showed 0% patency the next day after surgery (Figure 1, Figure 3 and Figure 4, Table 1). Despite the absence of blood flow, 12 months following surgery the biodegradable scaffold of PHBV/PCL-GFmix graft was almost entirely resorbed and showed no signs of calcification (Figure 3). On the other hand, GORE-TEX^®^ grafts displayed multiple foci of ectopic calcification both in the prosthesis wall and in adjacent connective tissue capsule as soon as 6 months following surgery (Figure 4). The calcification rate of GORE-TEX^®^ grafts varied from single barely noticeable foci of crystalline calcium in the graft wall to almost complete calcification of the prosthesis wall (Figure 4). 

**Figure 5:**
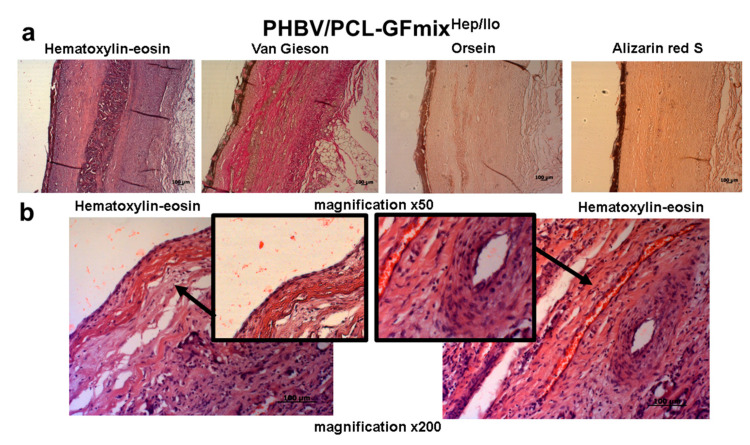
Representative images of PHBV/PCL-GFmix^Hep/Ilo^ grafts explanted 6 months following surgery. (**a**) Magnification 50×. (**b**) Magnification 200×. Scale bar = 100 μm.

PHBV/PCL-GFmix^Hep/Ilo^ grafts were patent in 62.5% animals 9 months following implantation, reducing to 50% at 1-year timepoint (Figure 1). All passable PHBV/PCL-GFmix^Hep/Ilo^ grafts developed the *de novo* vasculature with no signs of calcification (Figure 5a). Interestingly, the rates of PHBV/PCL graft resorption in sheep exceed those reported in the literature, that is 3 to 4 years for PCL and at least 5 months for PHBV [16,17]. In our previous studies, the degradation of PHBV/PCL grafts implanted into rats was also much longer as compared to this experiment [14,15]. In this study, PHBV/PCL-GFmix^Hep/Ilo^ grafts completely degraded only 6 months postoperation. Histological examination of explanted PHBV/PCL-GFmix^Hep/Ilo^ prostheses revealed scattered graft clusters surrounded by a newly formed vasculature (Figure 5b).

## 3. Discussion 

Arterial replacement is a treatment of choice for many patients with a vascular disease such as atherosclerosis. The best long-term results of bypass surgery are observed in patients implanted with autologous blood vessels such as the saphenous vein or internal thoracic artery; nonetheless, their availability is frequently limited due to multiple reasons including pre-existing vascular disease or vein stripping [2]. The development of novel biodegradable polymer grafts of small caliber may dramatically improve vascular disease management and increase the quality of life of patients with atherosclerosis.

In this report, we conducted a pre-clinical investigation to evaluate the patency of experimental PHBV/PCL grafts modified by a mix of growth factors VEGF, bFGF and SDF-1α (PHBV/PCL-GFmix), and additionally treated by antiplatelet agents and anticoagulants (PHBV/PCL-GFmix^Hep/Ilo^), upon their implantation into the carotid artery of sheep. Our previous research demonstrated an excellent performance of these grafts upon the implantation into rat abdominal aortas; however, the rat model may not be optimal for testing biodegradable vascular grafts due to rapid endothelization observed in these animals. Thus, we aimed to reinforce our findings using a sheep model, which is considered more aggressive than rat in terms of thrombosis and calcification, thus being more optimal for in vivo testing of cardiovascular implants [18,19,20].

Our first finding of significance is that carotid arteries of the sheep implanted with both experimental PHBV/PCL-GFmix and clinically approved GORE-TEX^®^ grafts display 100% thrombosis 24 h following surgery. In a rat model of abdominal aorta implantation, our PHBV/PCL-GFmix grafts remained fully patent for the entire 12 months of the study [14]. This dramatic difference between animal models is consistent with high rates of thrombosis in the sheep documented by others [18,20]. Synthetic grafts such as GORE-TEX^®^ are successfully used in the clinic to replace blood vessels with a diameter of >6 mm; however, their implantation into small-diameter arteries of lower blood flow velocity leads to rapid thrombosis, neointimal hyperplasia and the emergence of aneurysms [4,21]. Our results confirm the inability of 4 mm GORE-TEX^®^ grafts to support a minimal patency required for physiological functioning.

Despite this negative result, PHBV/PCL-GFmix grafts do not show any signs of calcination at 6 months following implantation, whereas 60% of GORE-TEX^®^ grafts undergo either mild or severe calcification, suggesting a high biocompatibility of the experimental PHBV/PCL blend. 

Unexpectedly, PHBV/PCL grafts (both GFmix- and GFmix^Hep/Ilo^-modified) exhibit rapid degradation and subsequent re-endothelialization when implanted into the sheep. This does not match with published data on PHBV and PCL grafts, documenting the in vivo degradation time of 3-4 years for PCL and at least 5 months for PHBV [16,17]. The observed discrepancy highlights the knowledge gap in understanding the biodegradation kinetics of polymer blends in different species.

Since a higher rate of thrombosis was expected for a sheep model, we attempted to reduce the thrombogenicity of PHBV/PCL-GFmix grafts by additionally incorporating antithrombogenic drugs heparin and iloprost into their surface. To do so, we coated grafts with PVP hydrogels by radiation-induced graft polymerization followed by incubation in heparin/iloprost mix. This approach enabled us to generate a highly porous and functionally active biodegradable vascular prosthesis serving as a backbone for the formation of a new vessel, and at the same time, to smoothen the pore relief of the tubular graft skeleton using the PVP coating and further improve the thromboresistance by incorporating heparin and iloprost. Six months following implantation, the resultant PHBV/PCL-GFmix^Hep/Ilo^ grafts demonstrate a primary patency similar to that of the sheep implanted with carotid artery autografts (62.5% vs. 87.5%, respectively). At the 1 year timepoint, 50% of PHBV/PCL-GFmix^Hep/Ilo^ sheep have fully patent protheses, indicating that modification with heparin and iloprost provides a significant advantage to the performance of PHBV/PCL grafts in large laboratory animals. Additionally, PHBV/PCL-GFmix^Hep/Ilo^ grafts were free of calcium deposits 12 months following surgery, while clinically approved GORE-TEX^®^ grafts show evidence of calcification already at the 6 month timepoint. 

Small-diameter blood vessels are characterized by lower blood flow velocity leading to poor passability of vascular grafts [4,21]. In our study, the blood flow velocity of sheep carotid arteries ranged from 56 to 85 cm/sec. Similarly, the blood flow velocity of a human carotid artery generally varies from 50 to 80 cm/sec. Thus, the sheep carotid artery implantation model is representative of human carotid artery, thus being an excellent model for small-diameter graft testing.

Findings presented in this brief report suggest that the biodegradable porous PHBV/PCL backbone is suitable for the formation of a new vasculature yet requires additional modification to reduce thrombogenicity. Thus, in this study we demonstrate a potential usefulness of the PHBV/PCL blend for developing biodegradable small-diameter vascular grafts for clinical use. We identified that PHBV/PCL grafts implanted into sheep display an optimal performance when additionally modified by anticoagulant drugs heparin and iloprost. However, more research should be done to optimize the current protocol of graft fabrication in order to improve the passability and reliability of these grafts. 

## 4. Materials and Methods

### 4.1. Graft Fabrication

Biodegradable vascular grafts were electrospun using a Nanon-01A instrument (MECC Co., Ltd., Moscow, Russia) at 23 kV voltage, solution feeding rate of 0.5 mL/h and 1.5 mm rotating drum diameter. The polymer blend contained 5% PHBV (Sigma-Aldrich, St. Louis, MO, USA) and 10% PCL (Sigma-Aldrich, St. Louis, MO, USA) dissolved in chloroform. The PHBV/PCL mix was further supplemented with human recombinant proteins VEGF, bFGF and SDF-1α (Sigma-Aldrich, St. Louis, MO, USA) at a ratio of 20:1 using the following regimen: the luminal part of the graft (approximately ^1^/_3_) was spun using a mix of PHBV/PCL and VEGF using a 27G needle to produce more nano-sized polymer filaments on the luminal surface; then, the electrospinning was continued using a 22G needle through which an mixture of the PHBV/PCL and bFGF and SDF-1α was delivered. The final concentration of each recombinant protein was 500 ng/mL. The characteristics of the resultant grafts were as follows: luminal diameter—4mm; length—40 mm; thickness—400 µm.

We incorporated VEGF into the luminal surface of grafts for better recruitment and adhesion of mature endothelial cells as well as their progenitors in order to form an endothelial monolayer. We additionally incorporated bFGF and SDF-1α into the graft wall for recruitment and maintenance of fibroblasts and smooth muscle cells as well as recruitment of bone marrow-derived mesenchymal stem cells and support of the newly formed blood vessels [22,23].

The resultant PHBV/PCL-GFmix grafts were further modified by antiplatelet agents and anticoagulants. Briefly, the luminal surface of grafts was hydrogel-coated by radiation-induced graft polymerization. Grafts were fully submerged in 5% polyvinylpyrrolidone (PVP, PanReac, Barcelona, Spain) ethanol solution for 30 min followed by air-drying for 24 h. Prostheses were then placed in argon-filled glass vials and irradiated at 50 kGy dose using a linear particle accelerator ILU-10 with a beam energy of 5 MeV 50 kW (Budker Institute of Nuclear Physics, Novosibirsk, Russia). The irradiated grafts were then submerged in a solution of 125 IU/mL heparin and 0.2 μg/mL iloprost in 0.1 M glycine-HCl (pH 2.6) for 30 min to ensure the binding of heparin and iloprost to PVP hydrogels. Grafts were then air-dried under sterile conditions.

### 4.2. Graft Implantation into the Carotid Artery of the Sheep

Female Edilbayev sheep weighing 42–45 kg with a carotid artery diameter of 3.9 to 4.2 mm evaluated by ultrasound dopplerography were used in the study. The study design was approved by the local ethical committee of the Research Institute of Complex Issues of Cardiovascular Diseases (issued 28.04.2016) and complied with Declaration of Helsinki (1996). 

Experimental groups included PHBV/PCL grafts modified either by GFmix alone (VEGF, bFGF and SDF-1α) or GFmix plus heparin and ilostat (Table 2). Sheep with carotid artery segments implanted as autografts were used as a negative control group with minimal thrombosis expected. Commercially available GORE-TEX^®^ vascular grafts (ST04010A, W. L. Gore & Associates, Inc., Medical Products Division, Flagstaff, AZ, USA) were used a control group (Table 2). 

Prior to graft implantation, sheep were anesthetized by intravenous injection of zoletil (Virbac, Hamilton, New Zealand) followed by intubation and mechanical ventilation with 4% sevoflurane (Abbott Laboratories, North Chicago, IL, USA). The heart rate, oxygen saturation and respiratory rate were closely monitored during the surgery. Sheep were intravenously administered with 1.5 g cefuroxime in 500 mL 0.9% NaCl during surgery.

After sterile preparation of the surgical field, an incision between the jugular vein and trachea on the left side along the anterior border of the sternocleidomastoid muscle was performed. Small branches of the jugular vein and carotid artery were ligated. A 7–8 cm long carotid artery segment was separated from the adjacent vagal nerve using a scalpel. Systemic heparinization using 5000 U intravenous heparin was performed. The carotid artery was clamped, and segments of 40 mm were resected at a 45° angle followed by graft implantation by end-to-end anastomosis using a continuous Prolene 6/0 suture (W8712, Ethicon, Somerville, NY, USA). For the negative control group, the resected carotid artery segments implanted back as autografts. The wound was closed using a Vicryl 2-0 suture (VCP326H, Ethicon, Somerville, NY, USA) followed by extubation. To avoid postoperative complications, all sheep received an intramuscular injection of 1.5 g cefuroxime every day for 5 days. The volume of intraoperative blood loss did not exceed 70–80 mL.

For graft patency examination, ultrasonography was performed at days 1, 5, 30, 90, 180, 270 and 360 following surgery.

### 4.3. Histological Examination

Explanted grafts were fixed in 10% neutral phosphate buffered formalin (BioVitrum, Moscow, Russia). Sample processing and histological staining was performed as described previously [9,10,11,12,13]. All grafts were stained for hematoxylin-eosin (H&E), Van Gieson, orsein, and alizarin red S.

## 5. Conclusions

Here we report the in vivo performance of our experimental PHBV/PCL-GFmix^Hep/Ilo^ biodegradable vascular grafts. Upon the implantation into sheep carotid artery, the grafts displayed a satisfactory patency 1 year following surgery (four out of eight sheep) as well as no evidence of calcification. The PHBV/PCL backbone completely degraded and was replaced by the neovascular tissue 6 months postoperation. Our findings indicate the high clinical relevance of PHBV/PCL-GFmix^Hep/Ilo^ grafts and set the ground for conducting clinical trials in the future. 

## Figures and Tables

**Table 1:** Primary patency, frequency of thrombosis and wall calcification across study groups.

Study Group	Primary Patency			Thrombosis			Calcium Deposition	
Timepoint	1 Day	6 Months	12 Months	1 Day	6 Months	12 Months	6 Months	12 Months
Carotid artery autograft implantation	7/8	7/8	7/8	1/8	1/8	1/8	0/8	0/8
GORE-TEX^®^	0/5	0/5	-	5/5	5/5	-	3/5	-
PHBV/PCL-GFmix	0/8	0/8	0/8	8/8	8/8	8/8	0/8	0/8
PHBV/PCL-GFmix^Hep/Ilo^	5/8	5/8	4/8	3/8	3/8	3/8	0/8	0/8

**Table 2:** Experimental groups of the study.

Study Group	Carotid Artery Autograft Implantation	GORE-TEX^®^	PHBV/PCL-GFmix	PHBV/PCL-GFmix^Hep/Ilo^
VEGF	N/A	-	+	+
bFGF	N/A	-	+	+
SDF-1α	N/A	-	+	+
heparin	N/A	-	-	+
iloprost	N/A	-	-	+
n	8	5	8	8
